# Three-Year Experience and Outcomes of Near-Early Internal Fixation for Femoral Neck Fractures in Pediatric Trauma Patients: A Retrospective Study

**DOI:** 10.7759/cureus.40049

**Published:** 2023-06-06

**Authors:** Pankaj K Mishra, Siddharth Jain, Mohammed Nadeem

**Affiliations:** 1 Department of Orthopaedics, All India Institute of Medical Sciences, Bhopal, IND; 2 Department of Orthopaedics, Gandhi Medical College, Bhopal, IND

**Keywords:** near early fixation, osteonecrosis of femoral head, delbet classification, paediatric fracture, fracture neck of femur

## Abstract

Background

The fractured neck of the femur in children is commonly caused by high-energy trauma, and despite its low incidence, complications are more frequent. Delayed presentation is not unusual in developing countries. The interval between injury and surgery is thought to be a critical factor in determining outcomes. This study aims to evaluate the effectiveness of "near early" internal fixation (24-72 hours) for fractured neck of the femur in children.

Methods

This is a retrospective observational study that analyzed complete case records from a period of seven years. Cases were classified according to the Delbet classification and outcomes were assessed using the Ratliff criteria with a minimum follow-up of three years.

Results

The study included 24 male and 11 female patients, with an average age of 11.28 years. The most common cause of injury was road traffic accidents. The fracture distribution in the study population was as follows: Delbet type II in 18 patients, Delbet type III in 10 patients, and Delbet type IV in seven patients. In our study, all patients underwent near-early fixation, meaning their fractures were fixed within 24-72 hours of injury. The average time for the clinical-radiological union was 8 weeks, and the most common complication was premature physeal fusion, followed by osteonecrosis.

Conclusion

In developing countries, where patients often experience delayed referrals and lack of awareness, near-early fixation (24-72 hours) of a fractured neck of the femur in children is a crucial option that holds significant value.

## Introduction

Femoral neck fractures in children are rare and account for less than 1% of all pediatric fractures [1]. This rarity is attributed to the relatively dense bone and thick periosteum in the femoral neck of children [2]. High-energy trauma is often associated with this type of fracture, and around 30% of cases involve another injury [3]. Although pediatric hip fractures are uncommon, their complications are more prevalent [4].

This study hypothesized that the prognosis of femoral neck fractures in children is inversely related to the working conditions and resource limitations that cause delayed presentation. The time from injury to surgical fixation is a crucial factor in determining the rate of complications, with early surgical intervention recommended. However, delayed management of pediatric hip fractures is common in developing countries due to factors such as ignorance, illiteracy, reliance on bonesetters, financial constraints, delayed referral services, and poor infrastructure [5,6].^ ^At our study center in India, fracture fixation usually occurs within 72 hours of injury. The study aimed to evaluate the outcomes of femoral neck fracture fixation in children within 24-72 hours of injury, known as "near early," at a tertiary care hospital in central India.

Despite extensive research, there is no existing study that exclusively examines the outcomes of pediatric femoral neck fractures managed within a trauma-surgery interval of 24-72 hours. As far as our knowledge goes, this is the first study to report the results of fixing pediatric femoral neck fractures as soon as possible within this timeframe (24-72 hours of injury), which we refer to as "near early.”

## Materials and methods

This retrospective observational study was conducted at a tertiary-level hospital in India. We reviewed the cases of pediatric femoral neck fractures treated at our facility from January 2010 to August 2016 and included those with complete case records, patients under the age of 16 who underwent surgery within 24-72 hours of injury, and a minimum follow-up of three years. We excluded cases of pathological fractures, those with less than three years of follow-up, and those operated on outside of the specified time frame. All cases were managed with either closed or open reduction and internal fixation using 4.5 mm partially threaded cannulated cancellous screws, except for one case that was fixed with a Moore pin and screw. Capsular decompression was not performed in cases where the closed reduction was achieved but was carried out in all other cases due to open reduction. Suction drains were not used in any cases, and no spica cast or de-rotation bar was applied postoperatively. Patient case records included age, sex, mode of injury, reduction/fixation method, union time, implant removal time, and any sequelae such as osteonecrosis, nonunion, premature epiphyseal fusion, and limb length equality. Follow-up assessments were conducted using Ratliff's method and categorized as good, fair, or poor (Tables 1, 2) [7]. The final follow-up X-rays were peer-reviewed for osteoarthritic changes, coxa vara, congruent hip joint, functional limitation, and avascular necrosis according to the Ratliff classification. Limb length discrepancy was measured using the indirect "block test" [8]. Continuous variables were expressed as mean ± standard deviation (SD), and categorical variables were expressed as the number of patients (%). Statistical analysis was performed using Microsoft XL 2007, with Pearson's chi-squared test used to compare values, and significance was set at p ≤0.05.

**Table 1:** Ratliff’s classification of osteonecrosis in pediatric hip fracture LEV: lateral epiphyseal vessels.

Grade	Description	Prognosis
1	Whole head involved, most common and severest type, involves all LEV	Worst prognosis
2	Head partially (segmentally) involved, involves few LEV	Poor prognosis
3	Rare but has a good outcome, physeal fracture followed by metaphyseal osteonecrosis (epiphysis sparing), and superior LEV involved	Good prognosis

**Table 2:** Clinico-radiological grading by Ratliff for the assessment of the outcome of the fractured neck of femur ROM: range of movement.

Criteria	Clinical	Radiological
Good	No pain or "ignores"; almost full ROM at hip; normal activity	Near normal or minimal deformity of the femoral neck in the radiograph
Fair	Occasional pain; ROM is more than 50%; mild avascular necrosis; avoidance of game	Severe deformity of the femoral neck
Poor	Disabling pain, severe avascular necrosis, ROM is less than 50%, activity is restricted	Degenerative arthritis and arthrodesis

## Results

The records from our orthopedic department from January 2010 to August 2016 included 39 patients who underwent surgical management for pediatric hip fractures. Of these patients, two were lost to follow-up, and two underwent surgery after the 72-hour trauma surgery interval. None of the patients underwent surgery within 24 hours of the trauma surgery interval, leaving a total of 35 patients for analysis. Among the patients, 5.7% (nine) had other associated injuries, which were addressed simultaneously in all cases of poly-trauma. No case of bilateral femoral neck fracture was present in our study population. Table 3 presents the demographic characteristics and outcomes of our study.

**Table 3:** Demographic depiction of our study RTA: road traffic accident;  PPF: premature physeal fusion; M: male; F: female; CRIF: close reduction and internal fixation; ORIF: open reduction and internal fixation.

S. No	Age/Sex	Displaced/Undisplaced	Delbet Type	Mode of Injury	Treatment	Complications	Results	Follow-up Period (Years)
1	11/M	Displaced	II	RTA	CRIF with screw	PPF, shortening	Fair	4
2	11/M	Displaced	IV	Trampling	CRIF with screw	Shortening, PPF	Fair	3
3	11/M	Undisplaced	II	Fall from height	CRIF with screw	PPF, shortening	Good	5
4	10/M	Displaced	II	RTA	ORIF with screw	Osteonecrosis, shortening	Fair	3
5	16/M	Displaced	II	RTA	CRIF with screw	Osteonecrosis, shortening	Poor	3.5
6	12/F	Displaced	IV	Trampling	ORIF with screw	Shortening, PPF	Fair	4
7	14/F	Displaced	III	RTA	CRIF with screw	PPF, shortening, osteonecrosis	Fair	5
8	14/F	Displaced	IV	Fall from height	CRIF with screw	Shortening	Good	4
9	14/M	Displaced	II	RTA	CRIF with screw	Shortening, PPF	Good	3
10	13/F	Displaced	II	RTA	ORIF with screw	Osteonecrosis, shortening	Poor	4.5
11	7/M	Undisplaced	III	RTA	CRIF with screw	Shortening	Good	5
12	11/M	Displaced	II	RTA	CRIF with screw	PPF, shortening	Good	3
13	13/M	Displaced	IV	RTA	CRIF with screw	Shortening, PPF	Good	4
14	15/M	Displaced	III	Fall from height	CRIF with screw	PPF, infection, screw cut-out, shortening	Fair	3.5
15	14/F	Displaced	II	RTA	CRIF with screw	Shortening	Good	3
16	11/F	Displaced	II	RTA	CRIF with screw	Shortening, PPF, osteonecrosis	Fair	4
17	14/M	Displaced	III	RTA	CRIF with screw	Osteonecrosis, PPF	Fair	4
18	12/M	Undisplaced	III	Trampling	CRIF with screw	PPF, shortening, osteonecrosis	Fair	5
19	9/M	Displaced	II	RTA	ORIF with screw	Shortening, PPF	Good	3.5
20	13/M	Displaced	II	RTA	ORIF with screw	Osteonecrosis, shortening	Fair	3
21	9/M	Displaced	III	Fall from height	CRIF with screw	Shortening, PPF	Good	3
22	9/M	Undisplaced	III	Fall from height	CRIF with pin	PPF, shortening	Good	3.5
23	9/M	Displaced	II	RTA	CRIF with screw	Osteonecrosis, PPF	Poor	4
24	8/F	Displaced	II	RTA	CRIF with screw	Shortening	Good	4
25	11/M	Displaced	IV	RTA	CRIF with screw	Shortening	Good	3.5
26	9/M	Displaced	II	RTA	CRIF with screw	Shortening, PPF	Good	3
27	12/M	Displaced	II	RTA	CRIF with screw	PPF	Poor	4
28	10/M	Displaced	III	RTA	ORIF with screw	Osteonecrosis	Fair	3.5
29	12/F	Undisplaced	II	Fall from height	CRIF with screw	Osteonecrosis, shortening	Fair	3
30	7/F	Displaced	III	RTA	CRIF with screw	Shortening	Good	3
31	13/F	Displaced	II	RTA	ORIF with screw	Osteonecrosis, shortening	Fair	4
32	10/M	Displaced	III	Trampling	CRIF with screw	PPF, shortening	Good	3
33	12/F	Displaced	IV	RTA	ORIF with screw	Shortening	Good	3.5
34	8/M	Displaced	IV	Fall from height	CRIF with screw	PPF, shortening	Good	4
35	11/M	Undisplaced	II	RTA	CRIF with screw	Shortening, osteonecrosis	Good	3

Our study included 35 participants with a male-to-female ratio of 24:11 and an average age of 11.28±2.28 years. The majority of injuries were caused by road traffic accidents in 24 patients (68.5%), followed by falls from height in seven patients (20%), and trampling in four patients (11.4%). Among the fractures, Delbet type II (transcervical fracture) was the most common (51.4%), followed by Delbet type III (cervico-trochanteric fracture) (28.5%) and Delbet type IV (intertrochanteric fracture) (20%). Closed reduction was achieved in 28 patients (80%), while the remaining seven patients (20%) required open reduction via the anterior approach to achieve anatomical reduction.

**Figure 1:**
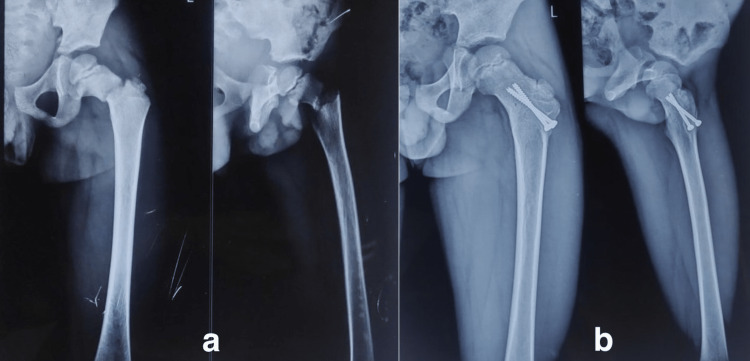
Eight-year-old female patient had a history of road traffic accident; (a) anteroposterior and lateral views showing Delbet type II fracture; (b) anteroposterior and lateral views showing good outcome at the follow-up.

**Figure 2:**
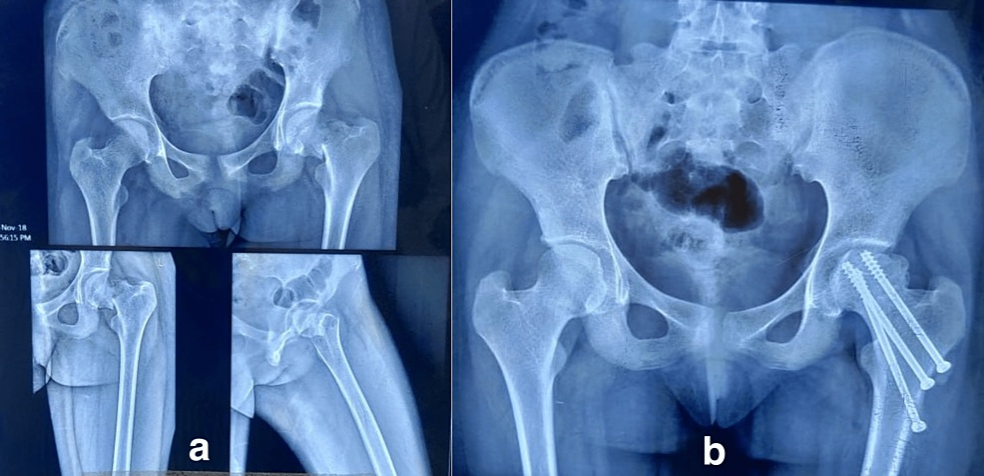
A thirteen-year-old female patient got injured in a road traffic accident. (a) The anteroposterior and lateral views show the Delbet type II fracture; (b) at 1 year of follow-up, she developed osteonecrosis (Ratliff type II) and had a fair outcome.

**Figure 3:**
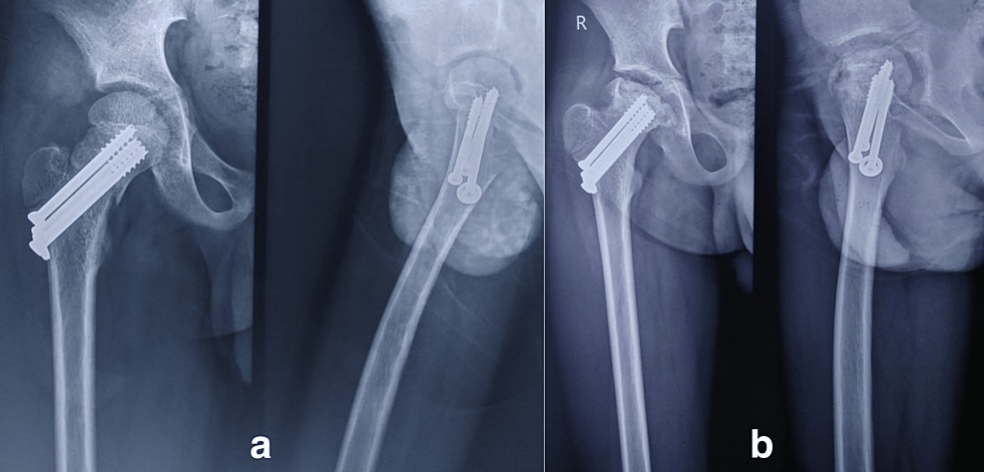
A twelve-year-old male had a road traffic accident. The (a) anteroposterior and lateral views show the Delbet type II fracture fixed with cannulated cancellous screw (b) and at the 1.5 years of follow-up, he developed osteonecrosis (Ratliff type I) and had poor outcome.

We followed up with patients for an average of 3.6±0.65 years or 39±3.64 months. Using Ratliff’s criteria, we categorized the outcome as "good" in 18 patients (51.4%), "fair" in 13 patients (37.1%), and "poor" in four patients (11.4%) (Figures 1, 2, 3). There were no instances of non-union, delayed union, or implant failure. Clinical-radiological union occurred at an average of 8±1.12 weeks. The most common complications were premature epiphyseal fusion in 57.1% of cases (20 patients) and osteonecrosis in 37.14% of cases (13 patients). The majority of osteonecrosis cases (69.2%) occurred in Delbet type II fractures (nine out of 13 patients), while the remaining cases (30.7%) occurred in Delbet type III fractures (four out of 13 patients). Ratliff type I osteonecrosis was present in seven patients (53.8%) and Ratliff type II osteonecrosis was present in the remaining six patients (46.15%).

**Figure 4:**
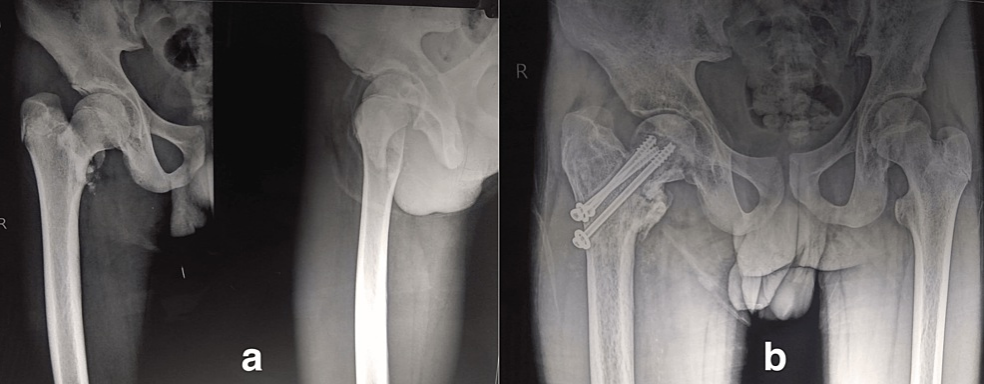
Fifteen-year-old boy had (Delbet type III) fracture due to a fall from height as shown in (a) anteroposterior and lateral views, managed by CRIF with cancellous screws. At the earliest follow-up visit, he revealed the history of early weight bearing. The patient was advised not to bear weight till further order. At eight months of follow-up, (b) anteroposterior view shows the superior screw cut-out and the patient presented with a pus-discharging sinus. CRIF: closed reduction and internal fixation.

Only one patient (2.8%) experienced an infection, which was associated with a superior screw cut-out, likely due to early weight bearing (Figure 4). Limb length discrepancy was found in 31 patients (88.5%), with an average shortening of about 0.6±0.22 centimeters. During follow-up, 17 patients (48.5%) had their implants removed for various reasons, including osteonecrosis, implant concerns, hardware problems, and infection.

We performed a statistical analysis of the rate of osteonecrosis in the context of patient age, displacement, and type of reduction (close/open). The rate of osteonecrosis was 11.1% in patients under 10 years of age (one out of nine patients) and 46.1% in patients aged 10 years or older (12 out of 26 patients), with no statistically significant difference between the two age groups (p=0.061). The rate of osteonecrosis in displaced fractures was 37.9% (11 out of 29 patients) compared to 33.3% (two out of six patients) in undisplaced fractures, with no statistically significant difference (p=0.832). The rate of osteonecrosis was 28.5% (eight out of 28 patients) for closed reduction and internal fixation and 71.4% (five out of seven patients) for open reduction and internal fixation, with a statistically significant difference (p=0.036). Therefore, the study concluded that among the factors analyzed (age, displacement, and type of reduction), only the type of reduction (close/open) had a significant impact on the rate of osteonecrosis (Table 4).

**Table 4:** Prognostic risk factors for osteonecrosis in our study

Parameters	Number of Patients	No. of Cases With Osteonecrosis	Pearson's Chi-squared Test (p-Value)
Age < 10 years	9 (25.71%)	1 (11.11%)	0.061
Age ≥ 10 years	26 (74.28%)	12 (46.15%)	
Displaced	29 (82.85%)	11 (37.93%)	0.832
Non-displaced	6 (17.14%)	2 (33.33%)	
No capsular decompression (close reduction)	28 (80%)	8 (28.57%)	0.036
Capsular decompression (open reduction)	7 (20%)	5 (71.42%)	

## Discussion

According to previous literature, Delbet type II (transcervical fracture) fractures are the most common, followed by Delbet type III (cervico-trochanteric fracture) and IV fractures (intertrochanteric fracture) [9]. In our series, we also found a similar distribution, with Delbet type II accounting for 51.4%, Delbet type III for 28.5%, and Delbet type IV for 20% of cases. Delbet type I fractures (trans-epiphyseal separation) are rare and were not encountered in our study.

Moon et al.'s meta-analysis showed a strong correlation between Delbet fracture types and the incidence of osteonecrosis, with rates of 28%, 18%, and 5% for Delbet type II, III, and IV fractures, respectively [10]. Dendane et al. reported a higher rate of osteonecrosis in Delbet type II fractures (44.44%) than in Delbet type III fractures (20%) or Delbet type IV fractures (0%) in their study [11]. In our study, osteonecrosis occurred in 67% of Delbet type II fractures, 33% of Delbet type III fractures, and 0% of Delbet type IV fractures, consistent with previous literature.

While some authors have suggested that the younger age group (below 10 years) has a positive effect on femoral head remodeling, others have suggested that although the severity of osteonecrosis is lower in younger patients, the rate of occurrence is not influenced by age group [10,12]. In our study, although we did not measure the severity of osteonecrosis, we found that it occurred predominantly (75%) in patients older than 10 years, with no statistically significant difference between age groups. Therefore, our limited study suggests that the rate of osteonecrosis is more likely to occur in older age groups.

The disruption of blood supply occurs during the time of the fracture, and fracture displacement causes vessel kinking or direct vessel tear due to fracture spikes. Therefore, the displacement of femoral neck fractures is strongly associated with osteonecrosis in children [13]. However, Kay et al. have suggested that displaced sharp fracture fragments can perforate the capsule and release the tamponade effect, reducing the intraarticular pressure [14]. In our study, we did not find a significant difference in the rate of osteonecrosis between displaced and undisplaced fractures. Therefore, we agree with previous research indicating a correlation between fracture displacement and osteonecrosis and suggest that fracture displacement (capsular rupture) does not have a forgiving effect on osteonecrosis.

Our study observed a lower incidence of osteonecrosis, coxa vara, and non-union, and we did not encounter any case of coxa vara and non-union. However, we did find premature physeal fusion in 57.1% of cases, which could be attributed to various factors such as delayed presentation, surgical technique, and osteonecrosis. In contrast to the larger studies conducted by Ratliff et al., Canale et al., and Lam et al., which primarily used conservative treatment methods, our study exclusively utilized surgical intervention and achieved significantly improved outcomes [4,7,9]. The incidence of coxa vara was 14%, 21%, and 32%, non-union was 33%, 6.5%, and 27%, and premature physeal fusion occurred in 20%, 62%, and 20% of cases in the Ratliff et al., Canale et al., and Lam et al. studies, respectively [4,7,9].^ ^The overall rate of infection due to surgical intervention for pediatric femoral neck fracture is around 1% [5].^ ^In our study, one case (2.8%) got infected and presented with a discharging sinus from the medial aspect of the thigh, possibly due to the small sample size. Nevertheless, we want to emphasize that careful soft tissue handling is crucial in preventing infections.

In 1928, Delbet first described and published pediatric femoral neck fractures in a French journal, and in subsequent years, Collona promulgated the Delbet classification, which is still widely acknowledged today for proximal femoral fractures in children [15]. Anatomical reduction and stable fixation are crucial for managing femoral neck fractures, and various treatment modalities have been described in the literature, including cast immobilization alone, closed reduction with cast immobilization, and closed/open reduction and fixation. However, a consensus treatment strategy for proximal femoral fractures in children is still evolving and the outcome of pediatric femoral neck fractures remains unpredictable, with complication rates ranging from 6% to 70% in the literature [16,17].

Osteonecrosis is a serious and notorious complication that has been reported in a wide range, from 0% to 92%, and various factors have been attributed to its incidence, including age, initial fracture displacement, fracture type, and fixation method [10,18,19].^ ^However, there are no records correlating the incidence of osteonecrosis with the adequacy of reduction and method of treatment. Initially, it was believed that the type of initial treatment had only a small effect on the incidence of osteonecrosis and that the trauma-surgery interval was the main influence. However, the fact that the incidence of osteonecrosis varies significantly among cases managed between different trauma-surgery intervals, regardless of the method of treatment, makes it difficult to account for a single reason for the broad range of osteonecrosis rates. The prognosis of osteonecrosis is critical because it has an inverse relation to the outcome, and the absence of treatment options makes it even more critical once it starts. Therefore, when treating pediatric femoral neck fractures, predicting the occurrence of osteonecrosis is of utmost importance.

The timing of surgical evacuation of hematoma in the management of displaced hip fractures in children has been a topic of discussion. Cheng et al. conducted a retrospective study of 14 patients and proposed an aggressive management protocol for displaced femoral neck fractures in children [20]. They managed all fractures by aspiration and internal fixation within 24 hours of injury and did not observe osteonecrosis or non-union in their series. Bukva et al. also advocated for early hip decompression to minimize complication rates [21]. However, subsequent studies did not support the efficacy of early hip decompression in reducing complications [22]. In this study, we did not decompress every hip but decompressed those hips that required open reduction.

Studies by Pforringer W et al. and MacDougall A et al. explained that osteonecrosis is an inevitable outcome due to disrupted vascularity from the initial trauma [23,24]. Studies by Swiontkowski et al. and Flynn et al. have shown that urgent (within 24 hours) open reduction and internal fixation can reduce the incidence of osteonecrosis in children [25,26]. They found that if the fracture is managed early (within 24 hours), pathophysiological events due to impaired blood supply of the femoral head are reversible. However, Bombaci et al. reported a high rate of osteonecrosis (54.6%) regardless of the timing of the procedure, suggesting that complications increased significantly when the procedure was performed after the first 24 hours and did not change significantly afterward [27]. In our study, we found a significant reduction in the incidence of overall complications and osteonecrosis with near-early fixation (within the first 24 to 72 hours) of fractured neck of the femur in children. We emphasize that “near early fixation,” even if early fixation (within the first 24 hours of injury) is not feasible, has a significant reduction in the rate of complications. However, the complication rate in this study was slightly higher than that in the past studies of early fixation (within 24 hours).

Table 5 presents a comparison of our study with other similar studies [21,28-30]. Bali et al. (36 patients) recommended that internal fixation as a conservative treatment was associated with a higher risk of loss of reduction [28]. They also advocated for anatomical reduction, even if it required open reduction over closed reduction [28]. Our study also supports the use of internal fixation to achieve anatomical reduction. Bukva et al. conducted a retrospective study of 28 patients and found that urgent treatment (within 12 hours of injury) and hip decompression significantly reduced the incidence of osteonecrosis (39.2%) [21]. Ju et al. conducted a comparative study of over 58 children with displaced femoral neck fractures and reported that open reduction resulted in a lower incidence of osteonecrosis and better outcomes compared to closed reduction and internal fixation [29]. This highlights the importance of anatomical reduction of displaced fractures, which our study also emphasized by having a low threshold for open reduction. Yaokreh et al. (16 patients) concluded that delayed and conservative management of femoral neck fractures in children can have negative outcomes and economic burdens in developing countries [30].^ ^Our study (35 patients) suggests that the “near early" fixation (within the first 24 to 72 hours) of pediatric hip fractures is forgiving and emphasizes that early fixation within 24 hours of injury is not the only solution.

**Table 5:** Enumeration of recent studies published on pediatric femoral neck fracture

Study	Year of Publishing	Total Enrolled Number of Cases	Average Age	Average Follow-up Period	Remarks of Study
Bali et al. [28]	2011	36	10 years	3.2 years	Internal fixation preferred over conservative treatment which carries high risk of failure of reduction
Bukva et al.[21]	2015	28	10.75 years	9 years	Good results of urgent treatment within 12-hour interval after injury
Ju et al. [29]	2016	58	9.1 years	3 years	Open reduction and internal fixation have promising outcome than closed reduction and internal fixation
Yaokreh et al. [30]	2018	11	9.4 years	3.6 years	Working condition has indirect relation to outcome, and the conservative treatment leads to various complications
Our study	-	35	11.28 years	3.25 years	The earliest possible surgical intervention should be done in all pediatric femoral neck fracture cases. Age and fracture displacement play important roles in prognostication.

## Conclusions

In pediatric femoral neck fractures, it is recommended to fix all cases as soon as possible to reduce the likelihood of complications. In developing countries, where patients often experience delayed referrals and lack of awareness, near-early fixation (24-72 hours) of a fractured neck of the femur in children is a crucial option that holds significant value. The findings of this study highlight the importance of near-early fixation (within a timeframe of 24-72 hours of injury) in reducing complications, with age and displacement serving as prognostic factors.

The limited number of participants (35) and the retrospective design of the study serve as its constraints. To draw a more conclusive outcome, prospective studies with larger study samples and a longer duration of follow-up are necessary.
